# SBA-15 Mesoporous Silica as Delivery Vehicle for rhBMP-2 Bone Morphogenic Protein for Dental Applications

**DOI:** 10.3390/nano12050822

**Published:** 2022-02-28

**Authors:** Dimitrios Gkiliopoulos, Ioannis Tsamesidis, Anna Theocharidou, Georgia K. Pouroutzidou, Evi Christodoulou, Evangelia Stalika, Konstantinos Xanthopoulos, Dimitrios Bikiaris, Konstantinos Triantafyllidis, Eleana Kontonasaki

**Affiliations:** 1 Laboratory of Chemical and Environmental Technology, Department of Chemistry, Aristotle University of Thessaloniki, GR-54124 Thessaloniki, Greece; dgiliopo@chem.auth.gr (D.G.); ktrianta@chem.auth.gr (K.T.); 2 Center for Interdisciplinary Research and Innovation (CIRI-AUTH), Balkan Center, 57001 Thessaloniki, Greece; 3 Department of Prosthodontics, School of Dentistry, Faculty of Health Sciences, Aristotle University of Thessaloniki, GR-54124 Thessaloniki, Greece; itsamesidis@auth.gr (I.T.); antheo@dent.auth.gr (A.T.); gpourout@physics.auth.gr (G.K.P.); evangelia.stalika@gmail.com (E.S.); 4 Laboratory of Advanced Materials and Devices (AMDeLab), School of Physics, Faculty of Sciences, Aristotle University of Thessaloniki, GR-54124 Thessaloniki, Greece; 5 Laboratory of Polymer Chemistry and Technology, Department of Chemistry, Aristotle University of Thessaloniki, GR-54124 Thessaloniki, Greece; evicius@gmail.com (E.C.); dbic@chem.auth.gr (D.B.); 6 Laboratory of Pharmacology, School of Pharmacy, Faculty of Health Sciences, Aristotle University of Thessaloniki, GR-54124 Thessaloniki, Greece; xantho@pharm.auth.gr

**Keywords:** mesoporous silica, SBA-15, bone morphogenic protein, rhBMP-2, periodontal ligament cells, protein delivery

## Abstract

(1) Background: A proposed approach to promote periodontal tissue regeneration in cases of peri-implantitis is the local administration of growth factors at the implant site. Recombinant human bone morphogenetic protein-2 (rh-BMP-2) can effectively promote bone regeneration and osseointegration and the development of appropriate carriers for its delivery is of paramount importance. The aim of the present study was to develop SBA-15 mesoporous nanoparticles (MSNs) with varying porosity, evaluate their biocompatibility with human Periodontal Ligament Cells (hPDLCs) and to investigate their effectiveness as carriers of rh-BMP-2. (2) Methods: SBA-15 type mesoporous silicas were synthesized via sol–gel reaction. The calcined SBA-15 samples were characterized by N_2_ porosimetry, Fourier transform–infrared spectrometry (FTIR), Scanning (SEM) and Transmission Electron Microscopy (TEM). Rh-BMP-2 loading and release kinetics were evaluated by UV spectroscopy. (3) Results: MSNs presented hexagonally arranged, tubular pores of varying length and diameter. Slightly higher loading capacity was achieved for SBA-15 with large pores that presented good hemocompatibility. MTT assay revealed no cytotoxic effects for all the tested materials, while SBA-15 with large pores induced a significant upregulation of cell viability at day 5. (4) Conclusions: SBA-15 MSNs may prove a valuable delivery platform towards the effective release of bone-inducing proteins.

## 1. Introduction

Mesoporous silica is a class of materials containing periodic arrays of pores, that have tunable size over the range of 2–50 nm [1]. Mesoporous silica nanomaterials (MSNs) gained a raised interest in various applications, such as catalysis [2], water treatment [3], drug delivery [4,5,6,7], etc., due to their production via varying and relatively simple methodologies [8], special structural features, extensive multifunctionality and facile functionalization [9]. SBA-15 (Santa Barbara Amorphous) is a well-ordered MSN with hexagonal pore arrangement and uniform pore sizes up to 30 nm [10,11]. As a large pore MSN, SBA-15 is an ideal carrier of large biomolecules (e.g., proteins), that combines high biomolecule loadings, biocompatibility, capacity for both hydrophobic and hydrophilic proteins with various isoelectric points (pI) and structural stability that protects proteins from premature degradation in body fluids [12,13,14,15]. Finally, although the properties of MSNs qualify them as ideal large biomolecule carriers, various aspects must be taken into account prior to their final application, such as their biodistribution and toxicity [9].

Regenerative medicine (RM) constitutes a promising field of medicine towards engineering or regenerating lost or damaged tissues, due to age or disease. Peri-implantitis is a pathological condition occurring at tissues around dental implants, characterized by inflammation in the peri-implant mucosa and progressive loss of supporting bone [16]. Local administration of antimicrobial drugs to counteract the changes of bacteria population at the implant site controlling peri-implantitis has been proven an effective alternative to systemic administration [17]. Apart from infection control, therapeutic strategies should include the employment of molecules that could guide cell growth and differentiation to promote functional tissue regeneration. There is a variety of growth factors, such as bone morphogenetic proteins, vascular endothelial growth factor, fibroblast growth factor etc., enrolled in stimulation of bone growth, synthesis of collagen and repair of fracture in in vitro and in vivo preclinical models and limited clinical studies also [18,19,20]. In 1965, Urist and his team were the first to successfully isolate BMP-2, the potential growth factor to induce osteogenic differentiation and bone formation [21]. In the late twenties, the US Food and Drug Administration finally approved the application of recombinant human BMP-2 (rhBMP-2) in the dental field for maxillary sinus grafting and bone-grafting procedures [22]. The delivery of rhBMP-2 into the defect is performed with synthetic bone-grafting materials. A major problem is that rhBMP-2 can easily become inactivated due to interaction with blood enzymes. For this reason, it is essential to develop appropriate carriers that ensure the efficient administration of protein without jeopardizing its activation state and evaluate their interactions with blood components. Mesoporous silica nanoparticles (MSNs) have shown capability of delivering drugs and proteins with high loading capacity varying release rates [23]. MSNs present extensive multifunctionality, based on their high specific surface and tunable pore size with high pore volume. Different types and synthetic routes of MSNs exist but the most applied formulations are those of SBA-15 and MCM-41. The SBA-15 type MSNs are comparable to MCM-41 type; however, they exhibit larger pore diameters and thicker walls, thereby presenting better hydrothermal and thermal stability than MCM-41 [23,24]. SBA-15 materials have large and highly ordered pores and uniform tunable channels, which vary in diameter from 2 to 30 nm making them advantageous materials as carriers. This is their greatest advantage in protein delivery [23,25,26] compared with the smaller pore diameter of MCM-41 type mesoporous materials, which have been preferably utilized in drug delivery applications [27]. They can provide sustained and localized release of therapeutically relevant factors. Slow acting molecules such as rhBMP-2 need to be delivered by a system that facilitates sustained release over a certain period, to allow bone healing and regeneration. Therefore, it is necessary to develop appropriate delivery systems for rhBMP-2 to extend functionality, by avoiding burst release. Considering that drug delivery systems are critical for the function of rhBMP-2, the aim of the present study was to develop SBA-15 mesoporous nanoparticles with varying porosity, evaluate their hemocompatibility and viability with human Periodontal Ligament Cells (hPDLCs) and to investigate their effectiveness as carriers of rhBMP-2.

## 2. Materials and Methods

### 2.1. Synthesis of SBA-15 MSNs

SBA-15 MSNs with varying pore size were synthesized via sol–gel reaction according to previously reported methods [28]. In a typical procedure, Pluronic P123 was added to 400 mL of aqueous HCl solution, pH 1.6, and the mixture was stirred until complete Pluronic P123 dissolution had occurred. TEOS was then added dropwise, and the mixture was stirred under different conditions for each SBA-15 product. In the next step, the mixture was hydrothermally treated in a polypropylene autoclave. The as-made silica was collected with filtration, washed with ethanol (1 wash) and deionized water (~3 L) and dried in the environment for several days. The mesoporous material was obtained via calcination in air. The molar ratio of the raw materials was the same for all SBA-15 products and equal to: TEOS (1)/P123 (0.018)/HCl (0.208). The specific conditions for each SBA-15 product are presented in Table 1.

### 2.2. Physicochemical Characterization of Mesoporous Silicas

The morphology of the synthesized mesoporous silicas was examined using field-emission scanning electron microscopy. The electron microscope that was used was a JEOL JSM-7610F Plus supported by an Oxford AZTEC ENERGY ADVANCED X-act energy dispersive X-ray spectroscopy (EDS) system (JEOL Ltd., Tokyo, Japan)

For TEM imaging, powder samples of mesoporous materials were dispersed in ethanol and a drop of the suspension was placed onto a Lacey Carbon Film (Agar Scientific Ltd., Essex, UK). Imaging and analysis of the samples were performed with an FEI Titan-cubed, Themis 300 Transmission Electron Microscope (TEM) equipped with multiple HAADF/ADF/BF STEM detectors and an FEI Super-X, 4-detector EDX system.

The calcined SBA-15 samples were characterized by N_2_ adsorption/desorption measurements at −196 °C, using an Autosorb-1 (Anton Paar GmbH, Graz, Austria) porosimeter. Mesoporous silicas were outgassed under vacuum at 150 °C for at least 20 h prior to the measurements. Specific surface area was calculated using the BET (Brunauer–Emmett–Teller) equation in the range of 0.05 ≤ *P/P*_0_ ≤ 0.25. Relevant pore size distributions were obtained from both the adsorption and desorption branches of the respective SBA-15 isotherms using the BJH (Batter–Joyner–Halenda) method. Total pore volume was determined in correlation to the adsorbed N_2_ volume at *P/P*_0_ = 0.99.

Fourier Transform–Infrared Spectroscopy (FTIR) was executed with a Perkin Elmer Spectrometer (Perkin Elmer Inc., Waltham, MA, USA). The measurements were carried out in the transmittance mode (400–2000 cm^−1^), with a resolution of 2 cm^–1^ and 32 scans. For the investigation of the FTIR measurements, KBr (Merck KGaA, Darmstadt, Germany) pellets were prepared under a maximum of 7 tons of pressure with SBA-15 nanocarriers, with a powder of the sample-to-KBr ratio of approximately 1:100.

### 2.3. rhBMP-2 Loading on Mesoporous Silicas

The rhBMP-2 solution (0.5 mg/mL) was prepared by dissolving the lyophilized protein (ProSpec-Tany TechnoGene Ltd., Ness-Ziona, Israel) using 0.02 M dilute acetic acid. An amount of 25 mg of each SBA-15 sample was subsequently added in 1 mL protein solution. The obtained dispersion was then placed into a mechanical shaker of a temperature-controlled environment, at 37 °C with a speed of 100 rpm for 24 h. The supernatant was collected via centrifugation at 9000 rpm for 5 min, and its absorbance was measured at 222 nm using a UV–Vis spectrophotometer (Shimadzu UV-1700, Kyoto, Japan) to indirectly determine the loaded amount of rhBMP-2 to the system. The protein quantification was based on a standard curve previously prepared at 0.0025, 0.005, 0.01, 0.025, 0.05, 0.1, 0.25, 0.5 ppm rhBMP-2. The precipitated SBA-15/protein system was dried at room temperature and preserved in a sealed vial at 4 °C. The loading experiments were performed in triplicate.

The protein loading content and the entrapment efficiency of rhBMP-2 protein in both SBA-15 materials were calculated according to Equations (1) and (2), respectively.

(1) $$Drug\;loading\;content\;\left(\%\right)=\frac{weight\;of\;drug\;in\;microparticles}{weight\;of\;microparticles}\;\cdot 100\;$$

(2) $$Entrapment\;efficiency\;\left(\%\right)=\frac{weight\;of\;drug\;in\;microparticles}{weight\;of\;drug\;fed\;initially}\;\cdot 100\;$$

### 2.4. In Vitro rhBMP-2 Release Study

An accurately weighted amount of each SBA-15/rhBMP-2 system was placed inside a dialysis tubing cellulose membrane (SERVAPOR^®^ dialysis tubing, MWCO 12,000–14,000) and into a Falcon^®^ polystyrene test tube (14 mL). An amount of 10 mL of PBS buffer solution (pH = 7.4) was then added. Release tests were performed at 37 ± 0.5 °C and the rotation speed was set at 100 rpm. At predetermined time intervals (0.25, 0.5, 1, 2, 4, 6, 8, 24 h), 3 mL of the aqueous solution were withdrawn from the release medium, and an equal amount of fresh PBS was added to maintain the sink condition. The samples were filtered and assayed for rhBMP-2 release by the same UV method, and the quantification was determined in reference to the standard curve described previously. In each experiment, the samples were analyzed in triplicate.

### 2.5. Isolation of Human Periodontal Ligament Fibroblasts (hPDLFs)

Cultured cells (80% confluence) established from human biopsies of periodontal ligament tissues from a healthy donor were resuspended in phosphate-buffered saline (PBS) and incubated for 30 min at 4 °C with a specific direct conjugated antibody to CD34, CD45, CD73, CD105, CD146 and STRO-1, in accordance with manufacturer guidelines. The study was approved by the Institutional Ethical Committee (#110/10-2-2021).

### 2.6. Biocompatibility

The cellular metabolic activity of hPDLCs in contact with SBA-15, unloaded and loaded with rhBMP2, was evaluated at days 1, 3 and 5. All MSNs (stock solution: 1 mg/mL) were disinfected with UV light for 90 min. Cells of passage 4 were seeded in 96-well plates (1 × 104 cells/well) for 24 h to attach. Cells were then exposed to different concentrations of MSNs (dilutions 12.5, 60 and 125 μg/mL) for 1, 3 and 5 days of incubation, and experiments were performed in sextuplicate. Evaluation of mitochondrial activity and, thus, cell proliferation was performed and calculated as previously described with the MTT assay [27]. Briefly after the reduction of the yellow tetrazolium salt (3-(4,5-dimethylthiazol-2-yl)-2,5-diphenyltetrazolium bromide) to purple formazan crystals by metabolically active cells and the use of DMSO as solvent, optical density was determined spectrophotometrically at a wavelength of 545 nm and a reference filter of 630 nm using a microplate reader (Epoch, Biotek, Biotek instruments, Inc, Winooski, VT, USA). The results are presented as an average% percentage in respect to the positive controls’ values. Statistical analysis was performed using *t*-test, with statistical significance set at *p* < 0.05.

### 2.7. Blood Sample Collection

Fresh erythrocytes were separated from whole blood collected from the Blood Donation Department of the General Hospital of Naousa, Greece. Blood donor confidentiality was wholly preserved. Good Clinical Practice guidelines and the Declaration of Helsinki were followed according to the Ethical Committee of the hospital’s (ID_233205920) approval of the study.

### 2.8. Hemocompatibility

The hemocompatibility of erythrocytes with the MSNs suspension (stock solution: 10 mg/mL) was determined as follows: RBCs were prepared in PBS in a final suspension consisting of 5% volume erythrocyte (final volume: 1 mL) (hematocrit: 5%). Diluted RBCs were treated with different concentrations of MSNs 0.06, 0.125, 0.250, 0.500, 1, 2, 5 mg/mL) for 60 min and 24 h of incubation at 37 °C (Thermomixer-Biosan). The supernatant of untreated RBCs was used as negative control (Ctrl-) and RBCs treated with lysis buffer were used as the positive control. All the samples were centrifuged at 2000 rpm for 1 min and a microplate reader (Thermo Scientific, Waltham, MA, USA) was used to measure the absorbance of hemoglobin release in the supernatant of treated samples. The absorbance value of hemoglobin at 541 nm was measured with the reference wavelength of 700 nm. The percent of hemolysis was calculated as previously described [27].

## 3. Results

### 3.1. Physicochemical Characterization of Mesoporous Silicas

**Figure 1:**
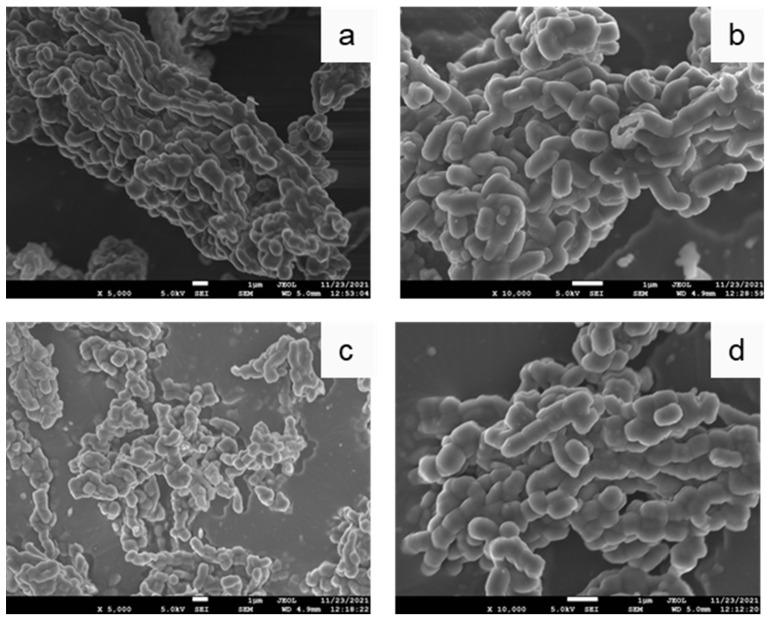
SEM images of the synthesized mesoporous silicas. (**a**,**b**) SBA-15 (8), and (**c**,**d**) SBA-15 (4).

The morphology of mesoporous silicas was examined with scanning electron microscopy. SEM images of SBA-15 materials that were synthesized under different conditions are shown in Figure 1. As evidenced, large pore SBA-15 (8) forms cylindrical clusters (Figure 1a), that consist of rodlike primary particles with 1.5 μm length and 0.7 μm width (Figure 1b). On the contrary, small pore SBA-15 (4) aggregates have irregular shape (Figure 1c) and consist of smaller elementary units with 1 μm length and 0.5 μm width (Figure 1d). The higher length of SBA-15 (8) primary particles, compared to SBA-15 (4), can be correlated to the higher synthesis temperature [29,30]. Moreover, differences in synthesis conditions strongly affect the kinetics of SBA-15 formation that, in turn, is correlated with the differences in the particle shape and size [31,32] of the two SBA-15 variants. 

**Figure 2:**
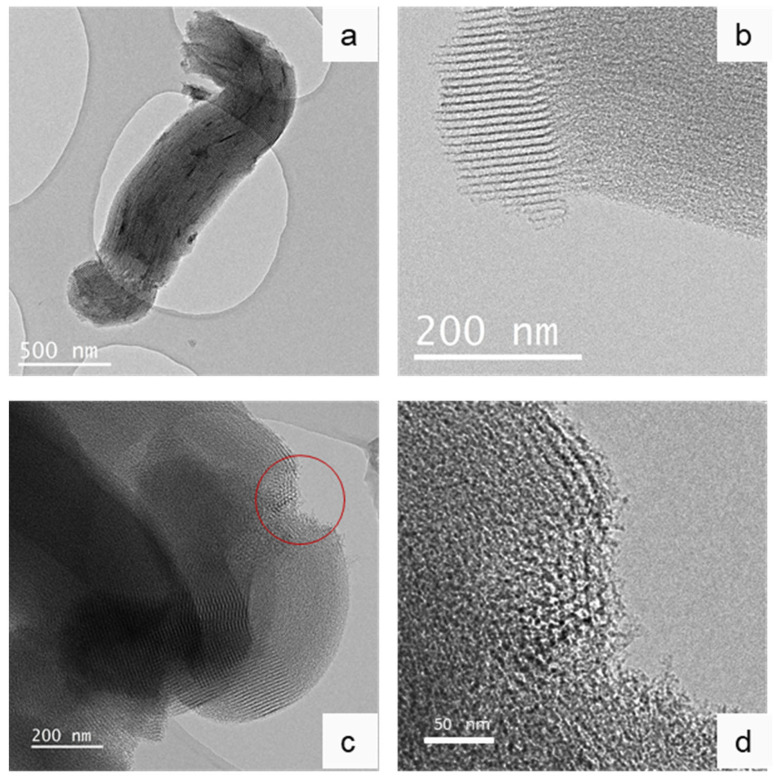
TEM images of SBA-15 (8) mesoporous silica. (**a**) primary SBA-15 (8) particle, (**b**) profile of parallel, tubular pores, (**c**) Hexagonal pore arrangement, and (**d**) magnification of the area inside the red circle of image (**c**).

TEM images of SBA-15 (8) are presented in Figure 2. As can be seen in Figure 2a, the tubular mesopores are parallel to the longitudinal axis of the particles and are extended from one end of the primary particle to the other, without any defects along their length. TEM can also be used to estimate the size of the pore diameter. The mean pore diameter, as it was measured from Figure 2b, equals 7.5 nm, which is close to the 8 nm estimation of physisorption tests (shown below). The typical 2D-hexagonal pore arrangement of SBA-15 mesoporous silicas can be seen in Figure 2d, which is the magnification of the area inside the red circle of Figure 2c. 

**Figure 3:**
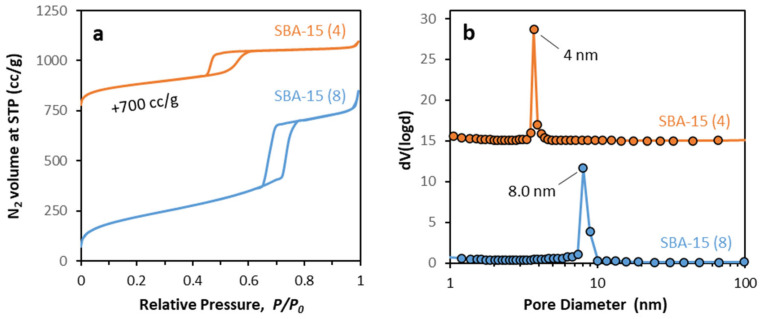
N_2_ porosimetry of SBA-15 mesoporous silicas, (**a**) physisorption isotherms, and (**b**) pore distribution analysis via BJH method.

The N_2_ physisorption isotherms of SBA-15 mesoporous silicas, measured at −196 °C, are shown in Figure 3a, while the physicochemical parameters obtained from the isotherms are presented in Table 2. According to IUPAC classification [33], SBA-15 (8) exhibit type IV isotherms with an H1 hysteresis loop showing steep capillary condensation in mesopores at high relative pressures (0.7 ≤ *P/P*_0_ ≤ 0.8), while SBA-15 (4) has a type IV isotherm with an H2a hysteresis loop that is typical of a pore architecture in which structure effects are important [34]. The steep distortion branch can be correlated either with pore-blocking/percolation or with cavitation-induced evaporation [35]. Furthermore, both materials exhibit micropore filling and interparticle condensation isotherm steps at *P/P*_0_ < 0.05 and *P/P*_0_ > 0.9, respectively. The pore distribution analysis results, as obtained with the BJH method, are presented graphically in Figure 3b. As can be seen, both SBA-15 samples exhibit sharp peaks at 8 and 4 nm, for SBA-15 (8) and SBA-15 (4), respectively, that correspond to well-formed, homogeneous pores with the respective pore diameters. 

**Figure 4:**
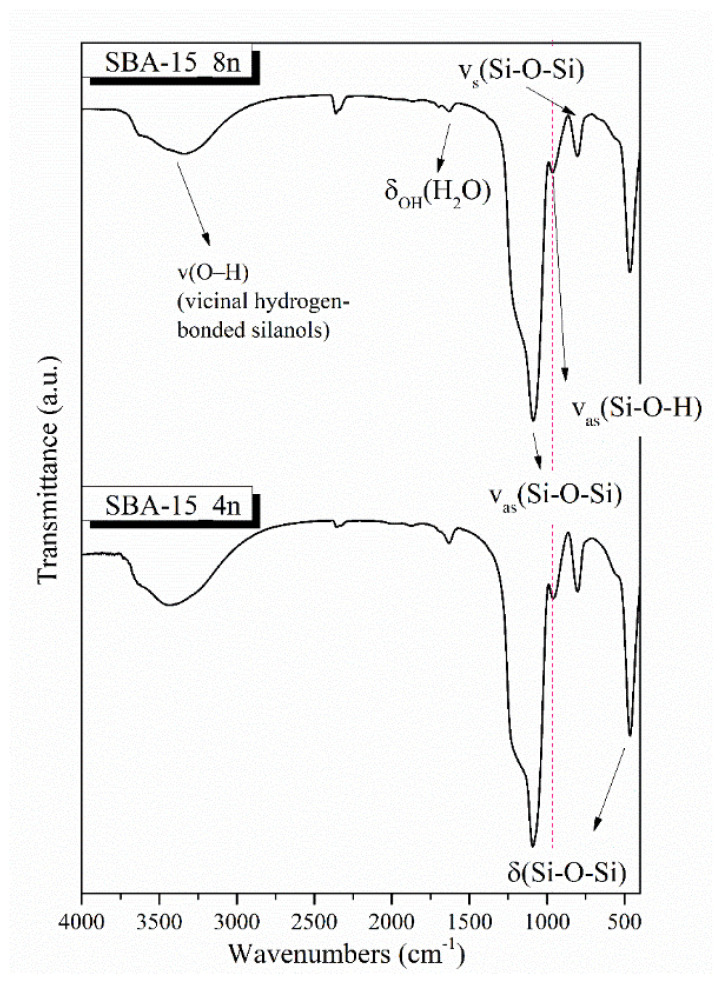
FTIR spectra of the synthesized SBA-15 MSNs.

The FTIR spectra of the synthesized MSNs are presented in Figure 4. The characteristic bands of amorphous silicate materials are presented in both SBA-15 MSNs. No remarkable changes were observed between the two materials of different pore size. More specifically, the broad peak located at around 990–1250 cm^−1^ corresponds to the Si–O–Si asymmetric stretching mode, the peak at 465 cm^−1^ is attributed to the vibration of the Si–O–Si bending mode and the peak at 805 cm^−1^ to the symmetric stretching vibration of Si–O. Additionally, the peak at approximately 965 cm^−1^ can be assigned to the Si-O stretching mode of the free silanol groups and the peak at around 1625 cm^−1^ indicates the presence of absorbed water on the surface of the samples [36,37,38]. The peak at around 3190–3600 cm^−1^ corresponds to the vibration of -OH bond, indicating the presence of vicinal hydrogen-bonded silanols [39].

### 3.2. rhBMP-2 Protein Loading and Release

The results of the drug loading content and the entrapment efficiency of rhBMP-2 protein in both SBA-15 materials are presented in Table 3. High entrapment efficiency was achieved in both cases, with a variation of ~10% higher for SBA-15 (8), most presumably owing to bigger pore size (volume and diameter wise). These findings are in agreement with Song et al. [40], who synthesized mesoporous calcium–silicon xerogels and found that the adsorption capacity of rhBMP-2 was double when mesopores were of 15 nm diameter compared with those of 4 nm. In the present study, low levels of loading were recorded because of the low amount of protein that was added in relation to MSN weight (1 μg of protein per 2.81 mg of MSNs). In other studies using spray-dried mesoporous bioactive glass microspheres [41] or pure mesoporous silicate nanoparticles [42], the protein amount was significantly higher in relation to MSN mass, thus yielding higher loading capacity. However, the exact dose of rhBMP-2 that can promote bone regeneration in different clinical applications without adverse effects has yet to be discovered, as different studies have reported controversial findings [43,44,45], increasing the reluctance and insecurity of clinicians to apply it [46,47]. It has not been proven that a high dose of rhBMP-2 can increase its efficacy [48], instead it can lead to increased inflammation [49,50], abnormal bone formation [51], root resorption [52], ankyloses [53], ectopic bone formation [50], gingival swelling [54], etc. Clinical results depend not only on the initial protein concentration but also to the type of carrier [55] and, in particular, its pore geometry and pore network, both affecting loading and release rate [56]. In the present study, we wanted to see whether these mesoporous materials were capable of entrapping this protein at low concentrations and if their pore size could have an effect on its loading and release profile.

**Figure 5:**
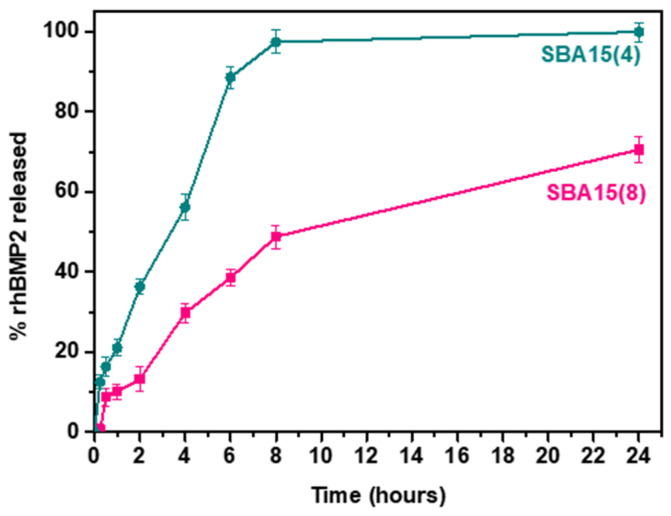
Amount (wt %) of released rhBMP-2 from SBA-15 MSNs at pH 7.4.

Figure 5 demonstrates the comparative in vitro release profiles of rhBMP-2 from the two mesoporous SBA-15 MSNs. As illustrated, ~10% of the adsorbed protein is released in the first half hour, followed by a biphasic release in both cases. The almost immediate release of the initial half hour, generally known as the “burst effect”, can be attributed to the protein adsorbed to the outer surface of mesoporous silica, while the remaining release points of both profiles can be correlated to the desorbed protein from the inner pore volume.

Considering the textural properties of SBA-15 materials, as well as the hydrodynamic size of rhBMP-2 (~2.57 nm), in the case of SBA-15 (4) the larger amount of protein is adsorbed in the interparticle pores of SBA-15 aggregates and close to the gates of tubular pores, which is why 95 wt % of the protein is released in the first 8 h. As for the remaining 5 wt % of the protein, it is slowly being released from the inner pore volume for the remaining 16 h of the measurement. On the other hand, SBA-15 (8) mesoporous silica has a larger pore diameter and volume compared with SBA-15 (4), while its interparticle micropore area and volume are smaller. Thus, a larger amount of adsorbed protein is expected to enter the mesopores. Considering the protein release profile, the amount of released protein in the first 8 h comes from both the mesopores and the interparticle space, while for the remaining 16 h the released protein comes from the mesopores.

The differences in the release rates can be attributed to several factors, such as the surface area and pore size of the mesoporous silica used, or the water permeability. Interestingly, in the case of SBA15 (8), although containing larger pores, the overall release process proceeds more slowly. This phenomenon could perhaps be attributed to the higher amount of protein loaded into the mesopores. As we have already experienced in a previous study with mesoporous nanoparticles [36], and according to Lai et al. [57], a higher initial loading concentration can lead to an over-packing of drug molecules into the inner pores, thus minimizing the dissolution rate. More specifically, although the rhBMP-2 loading and entrapment efficiency values were close for both SBA-15 samples, considering the mesopore and interparticle volumes, as well as the protein release profile, it can be safely assumed that the mesopore loading is higher for SBA-15 (8). Furthermore, rhBMP-2 of 2.57 nm hydrodynamic size could be better aligned in the 4 nm wide pore than the respective 8 nm wide pore, resulting in protein over-packing inside the SBA-15 (8) mesopores.

### 3.3. Evaluation of Biological Behavior of SBA-15 Loadedwith rhBMP-2

#### 3.3.1. Hemocompatibility Assay

**Figure 6:**
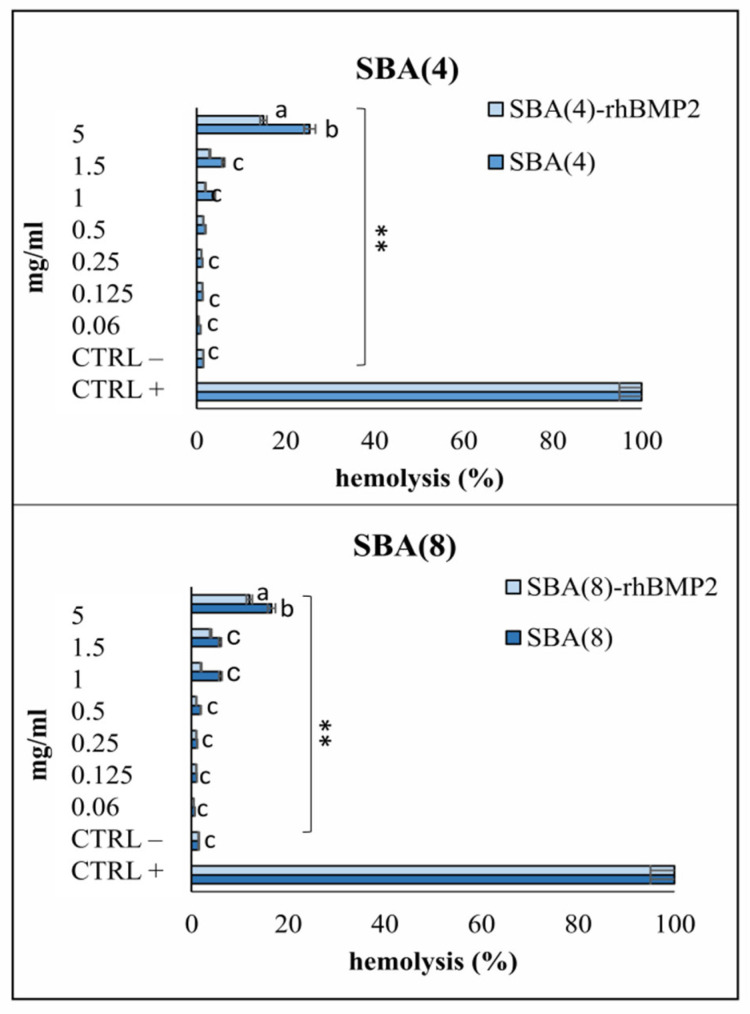
Hemolytic activity of unloaded and rh-BMP2-loaded MSNs after 60 min of incubation at 37 °C). ** indicates statistically significant difference (*p* < 0.001) between treated cells and untreated (controls), while different letters suggest statistically significant differences (*p* < 0.001) among concentrations.

Hemolytic activity is the most vital screening test, according to ISO10993–4 [58] parameters, as it evaluates the rupture of erythrocyte membranes in contact with the materials [59]. The hemolysis of the investigated formulations ranged from 1% to 25% depending on the concentration. As shown in Figure 6, SBA-15 (4) and SBA-15 (8) present hemocompatibility at concentrations lower than 1 mg/mL, which is a very high concentration. In this study, higher concentrations compared with those used in in vivo studies with animal models (0.06–0.12 mg/mL) [58,60] were tested to reveal the potential protection of MSNs to erythrocytes. SBA-15 (8) MSNs with a longer pore diameter present better hemocompatibility compared with those of SBA-15 (4) (*p* < 0.05). When MSNs were loaded with protein rh-BMP2, the hemocompatibility improved, especially at high concentrations (2 and 5 mg/mL). In the literature, it is well demonstrated that loading of nanoparticles and microparticles with pharmaceutical compounds (drugs, proteins etc.) decreases the hemolytic effect on healthy erythrocytes [58,61]. Zhao and his team [62] demonstrated that adsorption of large SBA-15-type MSNs (~600 nm) to erythrocytes induces hemolysis at concentrations > 20 μg/mL, in contrast with our study where SBA-15 type presented hemolytic effects only at high concentrations (concentration 0.5 mg/mL: 2% hemolysis for SBA (4) and 1.9% for SBA (8) MSNs, concentration 5 mg/mL: 25.4% hemolysis for SBA (4) and 16.7% for SBA (8)). Large pore SBA-15 MPs appeared [63] to enhance hemocompatibility and can be indeed considered less toxic compared with SBA-15 (4). The findings of the present study are in accordance with those of Ferraz et al. [64] who reported significant differences in hemocompatibility of alumina nanoparticles depending on their pore size. It has been reported that in large pores, proteins may be found at different conformational states, exposing different binding sites, thus yielding different interactions with blood cells [65].

Several parameters in material science have been postulated to be involved in the hemolytic activity of mesoporous nanoparticles, such as oxidative stress on the silica surface, denaturation of hemoglobin through electrostatic interactions with silicates and, in terms of surface charge, the amount of surface silanols. In the present study, there were no significant differences in terms of silanols, but the different textural characteristics and pore size may affect the continuity of the external surface layer of silanols [66], to a different degree. Other contributing factors to these minor differences in hemolytic profile may be the mixed shape and size of the MSNs and their aggregation state, as these parameters have been reported to affect endocytosis, toxicity [67] and hemocompatibility [66,68].

#### 3.3.2. Biocompatibility Assay

**Figure 7:**
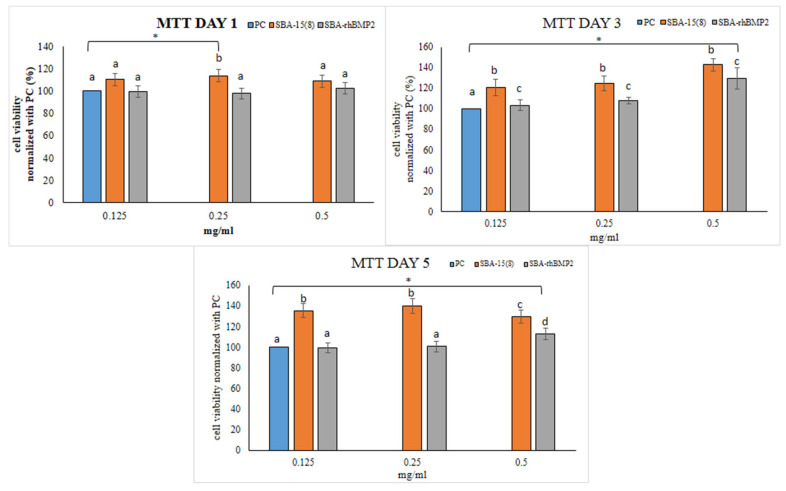
Cytotoxicity results of SBA-15 (8) MSNs at different concentrations (mg/mL). PC = plain cells without MSNs. The lines with * above bars indicate statistically significant differences (*p* < 0.05) of cell viability between control (plain, untreated cells) and cells treated with different concentrations of MSNs (controls), while different letters above bars suggest statistically significant differences (*p* < 0.05) of cell viability among the cells treated with different concentrations of MSNs. Same letters above the bars suggest that cell viability did not differ significantly among the specific MSNs and associated concentrations.

Once we demonstrated the hemocompatibility of both SBA-15 MSNs, we elected the optimum hemocompatible material to be further analyzed in terms of biocompatibility in contact with hPDLCs. The cytotoxicity assay revealed that unloaded SBA-15 (8) and loaded with rh-BMP2 did not present cytotoxicity at day 1, 3 and 5 (Figure 7). The biocompatible behavior was confirmed by the last day of incubation (day 5) and a statistically significant positive effect on cell viability was observed in the case of SBA-15 (8) at all concentrations in comparison with the untreated cells (*p* < 0.05). The most notable and statistically significant increase (*p* < 0.05) was reported for the hPDLCs exposed to unloaded SBA-15 (8). Loaded SBA-15 (8) MPs are biocompatible with hPDLCs without significantly promoting their proliferation rate. This phenomenon could be attributed to the effect of protein on cell differentiation that may appear as a low rate of cell proliferation. It has been reported that BMP-2 release from mesoporous silicate rods loaded with rh-BMP-2 (0.5 and 1 μg/mL) enhanced the ALP activity of bone marrow stem cells and induced early osteogenic differentiation at day 3 [69] compared with the control group. In the present study, STRO-1^+^ primary human PDL cells, well known for their osteogenic differentiation capacity (Supplementary Figure S1), were used. Τhe double negative CD34-, CD45- cells expressed STRO-1 at 97% and those cells were positive for CD146 at 98.2%; CD45 and CD34 expression was not observed on the cell surface of the 83.7% cultured cells population. Thus, a possible explanation of the lower proliferation rate of cells seeded with the protein-loaded MSNs is that their populations are reduced through autophagy degradation, which has been closely associated with early stages of differentiation evoked by the presence of protein. In a similar study, Rosenberg et al. [70] found no significant differences between BMP-2-loaded porous silicon carriers and an unloaded control, but both loaded and unloaded materials were biocompatible without differences compared with bone marrow mesenchymal stems cells alone. In any case, BMP-2-loaded mesoporous nanocarriers are biocompatible and, as an effective and safe rhBMP-2 dosage for bone regeneration has not yet been determined and the appropriate vehicle of protein delivery is still under investigation [71,72], this type of materials may prove a valuable delivery platform towards the effective release of bone-inducing proteins. Future areas of investigation include the optimization of release rate by functionalization and more efficient bonding of the protein within the pores, and cell culture studies, to verify the differentiation potential of these MSNs in vitro.

## 4. Conclusions

SBA-15 type mesoporous silicas were successfully synthesized, bearing hexagonally arranged, tubular pores with varying length and diameter. High protein entrapment efficiency was achieved in both cases, with a variation of ~10% higher for SBA-15 (8). Also, SBA-15 (8) appears to have better hemocompatibility compared with SBA-15 (4). MTT assay of the most hemocompatible SBA-15 (8) revealed no cytotoxic effects for all the tested materials. SBA-15 (8) induced a significant upregulation of cell viability at day 5.

## Figures and Tables

**Table 1:** Synthetic procedures of SBA-15 variants.

Synthesis Step	SBA-15 (8)	SBA-15 (4)
P123 dissolution in HCl (aq.) pH 1.6	Stirring at 38 °C until complete dissolution	Stirring at 38 °C until complete dissolution
TEOS hydrolysis and polymerization	Stirring at 40 °C for 24 h	Stirring at 35 °C for 1 h
Hydrothermal treatment	100 °C, 72 h	35 °C, 48 h
Product recovery	Filtration. Drying at T_room_	Filtration. Drying at T_room_
Calcination	550 °C, 6 h, 1 °C/min	550 °C, 6 h, 1 °C/min

**Table 2:** Physicochemical parameters of SBA-15 materials as obtained from N_2_ physisorption experiments.

Sample	Specific Surface Area	Pore Diameter (des.)	Total Pore Volume	Micropore Volume	Micropore Area	External Surface Area
	(m^2^/g)	(nm)	(cc/g)	(cc/g)	(m^2^/g)	(m^2^/g)
SBA-15 (8)	806	8	1.317	0.05	134	672
SBA-15 (4)	651	4	0.615	0.11	252	399

**Table 3:** Drug loading and entrapment efficiency of rhBMP-2 protein into SBA-15 MSNs.

Sample	Drug Loading Content (%)	Entrapment Efficiency (%)
SBA-15 (8)	1.8	89.2
SBA-15 (4)	1.6	77.5

## Data Availability

Data are contained within the article.

## Supplementary Materials

The following supporting information can be downloaded at: https://www.mdpi.com/article/10.3390/nano12050822/s1, Figure S1 Representative flow cytometric density plot of hPDLCs using as reference markers: C45-C34, STRO-1 and CD146. A total of 83.7% of the cells were double negative for the hematopoietic markers C45-C34, 97% of these cells expressed STRO-1 and 98.2% CD146, proving the stem cell properties of human periodontal ligament cells.
