# Supplementary material for: SBA-15 Mesoporous Silica as Delivery Vehicle for rhBMP-2 Bone Morphogenic Protein for Dental Applications
Source: Nanomaterials (Basel). 2022 Feb 28;12(5):822. doi: 10.3390/nano12050822 (PMC8912683; doi:10.3390/nano12050822)
Supplement: Supplementary file 1 [file nanomaterials-12-00822-s001.zip › nanomaterials-1601214-SI.pdf]

Supplementary Materials

SBA-15 Mesoporous Silica as Delivery Vehicle for rhBMP-2 Bone Morphogenic Protein for Dental Applications

Dimitrios Gkiliopoulos [1,2], Ioannis Tsamesidis [3], Anna Theocharidou [3], Georgia K. Pouroutzidou [3,4], Evi Christodoulou [5], Evangelia Stalika [3], Konstantinos Xanthopoulos [6], Dimitrios Bikiaris [5], Konstantinos Triantafyllidis [1,2] and Eleana Kontonasaki [3,*]

1 Laboratory of Chemical and Environmental Technology, Department of Chemistry, Aristotle University of Thessaloniki, GR-54124 Thessaloniki, Greece; dgiliopo@chem.auth.gr (D.G.); ktrianta@chem.auth.gr (K.T.)

2 Center for Interdisciplinary Research and Innovation (CIRI-AUTH), Balkan Center, 57001 Thessaloniki, Greece

3 Department of Prosthodontics, School of Dentistry, Faculty of Health Sciences, Aristotle University of Thessaloniki, GR-54124 Thessaloniki, Greece; itsamesidis@auth.gr (I.T.); antheo@dent.auth.gr (A.T.); gpourout@physics.auth.gr (G.K.P.); evangelia.stalika@gmail.com (E.S.)

4 Laboratory of Advanced Materials and Devices (AMDeLab), School of Physics, Faculty of Sciences, Aristotle University of Thessaloniki, GR-54124 Thessaloniki, Greece

5 Laboratory of Polymer Chemistry and Technology, Department of Chemistry, Aristotle University of Thessaloniki, GR-54124 Thessaloniki, Greece; evicius@gmail.com (E.C.); dbic@chem.auth.gr (D.B.)

6 Laboratory of Pharmacology, School of Pharmacy, Faculty of Health Sciences, Aristotle University of Thessaloniki, GR-54124 Thessaloniki, Greece; xantho@pharm.auth.gr

* Correspondence: kont@dent.auth.gr

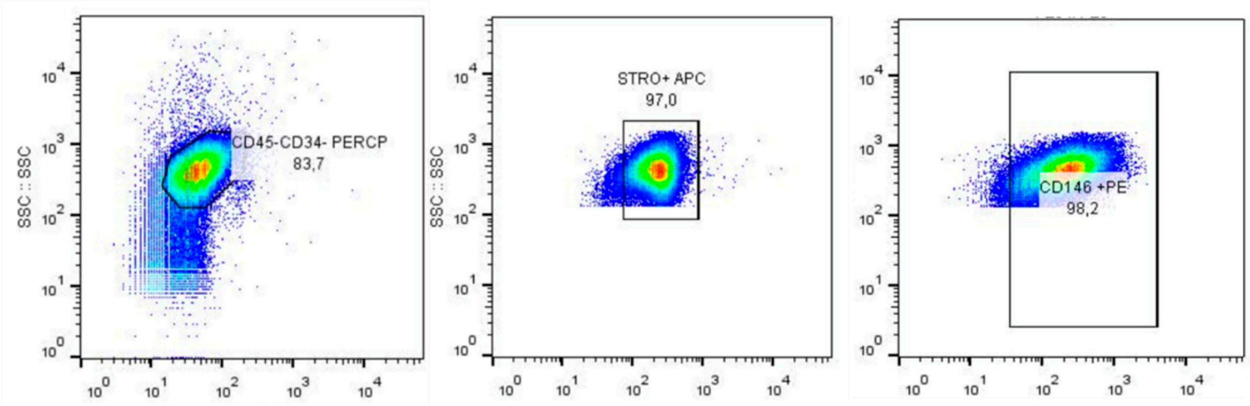

Figure S1. Representative flow cytometric density plot of hPDLCs using as reference markers: C45-C34, STRO-1 and CD146. The 83.7% of the cells were double negative for the hematopoietic markers C45-C34, 97% of these cells expressed STRO-1 and 98.2% CD146, proving the stem cell properties of human periodontal ligament cells.
